# The case for biocentric microbiology

**DOI:** 10.1186/1757-4749-1-16

**Published:** 2009-08-04

**Authors:** Ramy Karam Aziz

**Affiliations:** 1 Department of Microbiology and Immunology, Faculty of Pharmacy, Cairo University, 11562 Cairo, Egypt

## Abstract

Microbiology is a relatively modern scientific discipline intended to objectively study microorganisms, including pathogens and nonpathogens. However, since its birth, this science has been negatively affected by anthropocentric convictions, including rational and irrational beliefs. Among these, for example, is the artificial separation between environmental and medical microbiology that weakens both disciplines. Anthropocentric microbiology also fails to properly answer questions concerning the evolution of microbial pathogenesis. Here, I argue that an exclusively biocentric microbiology is imperative for improving our understanding not only of the microbial world, but also of our own species, our guts, and the world around us.

## Prologue

*"Despite our monumental achievements in philosophy, technology and the arts, to bacteria humans are no more than an organic mass to be utilized for growth and reproduction."*

***E.V. Sokurenko, et al. 1999 Trends in Microbiology***[1]

## Introduction: Two irreconcilable worldviews

Scientific revolutions involve paradigm shifts in the scientific community's worldviews, and the swiftness of such revolutions depends largely on the willingness of scientists to adopt novel ideas and perspectives. According to the science historian and philosopher Thomas Kuhn, "normal science" advances by the accumulation of data and findings that fit into an existing paradigm reflecting a particular worldview whereas scientific revolutions stem in response to "crises" caused by anomalies in normal science that need to be resolved[2]. These revolutions, according to Kuhn, often erupt as unaccepted ideas that eventually lead to paradigm shifts and are initially resisted by the community before becoming the new norms[2]. Indeed, unusual scientific ideas are often resisted by peer reviewers, funding agencies, and journal and book editors – the gatekeepers of "good science." There is hardly a better contemporary example than the struggle to establish *Helicobacter pylori *as an etiologic agent of gastritis and peptic ulcers[3], a landmark discovery that was resisted for year[4].

A common theme in major scientific revolutions (e.g., those ignited by Kopernik-Galileo, Darwin, and Einstein) is the decentralization of our worldview from anthropocentrism outward. Take, for example, the painful and controversial change in how humankind perceived the Earth's position in the universe. A paradigm shift from a universe revolving around planet Earth to a universe where Earth is one of several celestial bodies revolving around a star among a myriad stars was very slow to establish. Likewise, a paradigm shift in *Homo sapiens*' position among life forms – from being considered a biologically privileged species to a member of a cellular world that belongs to a universal tree of life[5] – is still the subject of endless debates, even in some "scientific" forums.

Surprisingly, although microbiology is a relatively modern science, it has not escaped the anthropocentrism associated with classical sciences like astronomy and physics. Since its birth, microbiology has been associated with human health and human interests (e.g., cheese, yogurt, beer, wine, pickles, and lately fuel). Its very name "***micro***biology" reflects an anthropocentric attitude, implying that because humans cannot see them, microbes are smaller than *normal*. Needless to say, this arbitrary nomenclature does not properly depict the biosphere. Biology is mostly microscopic[6]; humans, other macroscopic animals, and large plants are the exception[7]. The fact that human eyes have a limited visual range should not prevent humans from embracing a realistic view of nature. Nevertheless, research institutions and funding agencies give priority to the study of microbes that interact with human health, those that produce energy, or those that improve the taste and yield of human food, largely ignoring the majority of 4–6 × 10^30 ^estimated bacterial and archaeal cells on Earth[8].

Ten million years ago, there were no humans. One hundred million years ago, there were no mammals. Yet, members of the major bacterial and archaeal phyla had been thriving for thousands of millennia[9,10]. Thus, to imagine that the raison d'être of pathogenic or opportunistic bacteria is to survive by "harming" their host is simplistic, to say the least. The alternative viewpoint, which remains surprisingly uncommon in scientific literature and textbooks, is that some bacteria that had been stranded in the human body were driven to gradually evolve and adapt to such a hostile environment[11,12].

In this *Commentary*, I am openly claiming that current microbiology is contaminated with anthropocentric convictions (Table 1), many of which are irrational and negatively affect the objectivity of this science. Instead, I suggest that an exclusively *biocentric *microbiology (Table 1) is imperative for a proper understanding not only of the bacterial world, but also of the bacterial interactions with our species and other ecosystems on our planet.

**Table 1:** Some major differences between the anthropocentric and biocentric views of microbes

**The anthropocentric view of microbes**	**The (micro-) biocentric view of microbes**
Humans – being more complex, more sophisticated, and more important than microorganisms – are the center of attention.	Humans and microorganisms are cellular, nucleic acid-based life forms that struggle to survive and disseminate their nucleic material. They both deserve equal attention, and so do other acellular nucleic acid-based forms (e.g., viruses).

According to their effect on human health and lifestyle, microbes are classified into:	Humans and microbes share many ecosystems. Their interests converge or diverge, and their interactions include symbiosis as well as mutual killing. To microbes, humans also represent an ecosystem that they use as a relatively safe (?) haven and a source of nutrition. For this purpose, microbes do whatever it takes to better survive and disseminate.
- those that are useful (e.g., generate vitamins, food, and fuel) and need to be exploited for the common good of humankind	
- those that are harmful (e.g., cause diseases or spoil food) and need to be controlled and exterminated if necessary	
- those that do not interfere with humans	
The first two classes, albeit a minority among microbes, are the best studied and are preferred by funding agencies.	

Because humans' energy and resources are limited, anthropocentric microbiology focuses on microbes of interest to humans.	Biocentric microbiology implies that the thorough study of all bacteria, archaea, and other eukaryotic microbes (or representatives thereof) is imperative for the understanding of every single microbe, including those of direct interest to humans.

## The *status quo*: How anthropocentrism distorts microbiology

One prominent consequence of anthropocentrism is the artificial separation between medical and environmental microbiology. While the historical and practical reasons for such separation are understandable, they do not justify maintaining the *status quo*. Nowadays, the consideration that human or mammalian ecosystems – and their microbiota – are significantly distinct from other ecosystems, like soil, salt lakes, hot springs, or sediments, is scientifically tenuous. In my opinion, this separation has weakened both disciplines, depriving environmental microbiology of deserved attention, funding, and technological advances, and depriving medical microbiology of an eco-evo perspective[13,14]. Fortunately, the emerging field of metagenomics has successfully crossed the medical-environmental barrier, and it is becoming increasingly common to read that the mouth and gut microbiomes [15-17], for example, are being analyzed and described similarly to those in the soil [18] and other habitats[6].

Anthropocentrism has also had mostly negative impacts on the fields of taxonomy and phylogeny. Every taxonomic group to which *Homo sapiens *belongs has been historically inflated at the expense of other taxonomic groups, which have been collapsed together or totally ignored. It is not surprising then that the current tree of life has only been formulated about 30 years ago when the domain *Archaea *was established[19], and it is possible that novel major branches or even domains of life are still to be discovered[20]. Human-centered tendencies can be found in all early classification systems from those dividing living organisms into humans, animals, and plants to those dividing them into three or five kingdoms. Even the currently popular distinction between eukaryotes and everything else (i.e., prokaryotes) is criticized [21] and debated[22,23]. If you think about it, it is somehow arrogant to consider cells with additional membranes around their nucleic acids as *truly *(Greek. *eu*) nucleated, while those that have different membrane organization as primitive. This distinction is clearly an artifact of how humans drew analogy between fruit kernels and cell components that they called the nuclei. In the 21^st ^century, as we understand the differences between fruits and cells, the definition of a prokaryote is evolving[24].

Another sign of anthropocentrism's influence on microbiology is the distorted view of microbial pathogenesis, a view that artificially separates pathogenesis from other forms of adaptation[25,26]. A pathogenic bacterial lifestyle, bacteriocentrically speaking, should not be regarded as different from the behavior of a colony of *Homo sapiens *camping in a forest, exploiting some of the forest's resources, and leaving wastes that cause damage to that habitat. In fact, the current state of the global environment qualifies humans as the major pathogens of planet Earth. Just as an example among many, humans were recently described as co-pathogens of coral reefs[27].

In an anthropocentric world, the microbial virulence strategies include mechanisms dubbed immune evasion, invasion, and toxigenesis. In a biocentric world, much closer to reality, the same strategies can be considered as defense, nutrition seeking, and excretion mechanisms, respectively[28]. Who attacks whom, that is the question! Indeed, the fact that we, humans, have more bacterial cells than our own cells has prompted the rhetorical question, "who parasitizes whom?" [29]. An anthropocentric question such as "why do bacteria produce immunogenic molecules that alert the host to their presence, and then invest much energy to regulate and diversify these molecules to escape the host's immunity?" makes little sense. A bacteriocentric reciprocal question would be "why does the human host keep producing molecules that bind and deactivate the colonization factors that are essential to our (i.e., the bacterial) survival?" Obviously both questions are equally subjective, but role reversal may help humans realize their own subjectivity in dealing with microorganisms.

Another question that anthropocentrism cannot answer is why bacteria like staphylococci and clostridia secrete toxins in food items that would poison humans who ingest them without offering the bacterial cells any survival advantage in those humans. This and similar questions about the origin of toxigenesis can only be answered in an eco-evo context, according to which these toxins may be viewed as metabolic waste products that need to be excreted, as preemptive competition factors against other bacteria (e.g., bacteriocins and lantibiotics), or as antipredation defense mechanisms against protists[10].

By thinking outside the anthropocentric box, microbiologists answered a similarly puzzling question concerning the pathogenesis of the legionellae. These bacteria have not infected humans until the 20^th ^century, with the widespread use of showers and air conditioners[28]. They have certainly not been evolving for millions of years "in anticipation" to adapt to human macrophages. Instead, the pathogenesis of these aquatic bacteria could be explained as an adaptation to the macrophage-like protozoan predators, in whose vacuoles they evolved intracellular survival strategies[30].

One final example of anomalies arising from the anthropocentric contamination of our microbiology relates to the nomenclature of microbial proteins. For example, a bacterial protein was named Mac because of its similarity to a human macrophage receptor [31] and a bacterial genetic locus was called the locus of enterocyte effacement because it encodes gene products involved in damaging intestinal cells[32]. These names, and many more, may be accurately describing phenotypes associated with the expression of these proteins; however, they complicate the current efforts for automated genome annotations [33] because they fail to explain the functions of homologs of these proteins in organisms that never had a human encounter. Instead, annotations that are more biologically relevant should use controlled vocabulary related to the biochemical or structural properties of these proteins.

### Thinking like a microbe

*Attempts, in print and online, have been made to "think like a microbe" or describe the microbial world from a microbial perspective. For example, in *A Field Guide to Bacteria, *Betsey Dexter Dyer defends bacteriocentricity and tries to put herself "in the place of bacteria and observe the world as they observe it" *[7]*! To describe how this approach was taken too far, she tells the story of a friend who asked her advice on dealing with food poisoning. She writes, "I found that I was unable to properly sympathize with him (the human host) but instead came down quite strongly on the side of his intestinal bacteria, which, after all, were experiencing an invasion and were being dislodged from their habitat and deprived of their usual nutrients." (Ref*[7], *pages 6–7)*

*Another book that is even more bacteriocentric (*The Other End of the Microscope: the Bacteria Tell Their Own Story[34]) *describes a gathering, in which bacteria portray themselves more objectively, express their discontent with how they are named by humans, and finally suggest new names for the species *Homo sapiens.

*In the science blogosphere, addressing a mixed audience of scientists, scholars, students, and innocent bystanders, there are also attempts where the authors "impersonate" microbes, and let bacteria and viruses present themselves to the world (e.g*., Adopt A Microbe, ).

## Paradigm shift: How biocentrism can improve microbiology

Biocentric microbiology helps us better understand microbial pathogenesis. Classifying microbes into friends and foes, useful and harmful, often diverts us from recognizing the main goal of every microbe, which is no different from the main goal of every other organism: survival [28]. Host-associated microbes, including gut pathogens – the focus of this journal, would do whatever it takes for niche adaptation, defense against the immune system, and maximal dissemination. If some of their excreted/secreted proteins or metabolic byproducts represent a "conflict of interest" with human's multicellular tissues and organs, and if this conflict of interest offers the bacteria selective advantage over competing life forms around them (including human cells, other bacteria, or even peers) they will retain these proteins and use them, thus adopting what we call a pathogenic lifestyle. If their defense strategies necessitate hijacking human cells to hide in them and exploit their nutritious resources, again these "anti-human" traits will be selected, propagated, and possibly shared with other species via horizontal gene transfer. If, on the other hand, their survival necessitates killing or feeding on their parent, sibling, or daughter cells, they will resort to cannibalism and fratricide[35]. Before you call the latter mechanisms cruel or abhorrent, think about human cells that continuously commit fratricide and suicide during the processes of growth, neoplasia, and immune surveillance[36].

Biocentric microbiology will particularly benefit microbial genomics, phylogenomics, and-consequently – evolutionary biology. The focus on sequencing genomes of few bacterial phyla that interest *Homo sapiens *has led to a skewed representation of the tree of life [37,38] and a distorted view of the microbial world. Filling the gaps in the tree of life by sequencing genomes of more diverse bacterial and archaeal taxa will reveal many missing links and will fine-tune our understanding of metabolic networks. Metagenomic analysis will particularly benefit from a fairly represented tree of life. Currently, large fractions of sequenced metagenomes lack homologs in known databases and are thus uninformative[39]. With genome sequences from more diverse life forms, the informative sequences in each metagenomes are expected to multiply[40,41].

I also argue that biocentric microbiology will advance fields related to human health, including diagnostics, immunoprophylaxis, and therapeutics. The classical example of how diagnostics have benefited from environmental microbiology is the development of polymerase chain reaction (PCR)-based microbial identification tools. PCR is now essential in identifying and quantifying many human pathogens, and is sometimes the only reliable diagnostic method. This technology owes its speed and reliability to a bacterium that is totally out of anthropocentric microbiology's scope of interest: *Thermus aquaticus*.

In antimicrobial chemotherapy, the paradigm is shifting from screening different natural and synthetic products for drug *candidates *to screening microbial genomes and proteomes for drug *targets *that are essential and specific to bacteria. The conceptual shift here is from focusing on a tool to "kill the bad bugs" to a gene-product or a molecule without which the bacterial cell cannot survive. The same can be said about reverse vaccinology, the successful strategy that has revolutionized immunoprophylaxis. This strategy starts with the bacterial genome (bacteriocentric) to predict candidate immunogenic proteins rather than screening the sera humans or animals for antibodies against different bacterial proteins (usually those that can be purified from cultured bacteria) [42].

## Conclusion

At the end of this *Commentary*, having made the case for biocentric microbiology, I emphasize that I am not calling for self-hating ecocentric extremism. Human health is undoubtedly precious, and humans have every right to fight infectious diseases that kill and debilitate hundreds of million members of their species. However, when it comes to retaining the objectivity of science, a fundamental pillar of the scientific method, the boundaries between the disciplines of infectious diseases and microbiology should be made and kept clear. Microbiology should remain faithful to the study of its main subject: microbes. In doing so, it should equally focus on all microbes regardless of their interaction with humans.

### Open questions

*This *Commentary *is not intended as a comprehensive review of literature but as a primer for starting a discussion. Readers are encouraged to debate the ideas proposed here and to use the online discussion tools for commenting on the article. Below are some questions for such purpose*.

1) To what extent do you think that current microbiology is anthropocentric? What other signs of anthropocentrism can you detect that negatively affect this scientific field?

2) Do you agree that microbiology should be exclusively biocentric? Why or why not?

3) How can anthropocentrism help microbiology (other than to use human interests to get funding agencies' attention and support)?

4) How can "thinking like a microbe" be used as an efficient tool for microbiology education? What are the dangers of anthropomorphizing microbes?

## Competing interests

The only competing interest I feel obliged to declare is my belonging to the species *Homo sapiens*, mentioned in this article. My views are thus, inescapably, anthropocentric.

## Author's information

Ramy K. Aziz received his Ph.D. in microbiology and immunology from the University of Tennessee, Health Science Center, Memphis, TN, USA, and is currently a lecturer of microbiology and immunology at the Faculty of Pharmacy, Cairo University, Cairo, Egypt. His research interests include microbial pathogenesis, host-pathogen interactions and immunogenetics, microbial and phage genomics, and bioinformatics. In addition, he is interested in science blogging, open-access publishing, and undergraduate education.
